# Work-related violence and inconsistent condom use with non-paying partners among female sex workers in Adama City, Ethiopia

**DOI:** 10.1186/1471-2458-13-771

**Published:** 2013-08-23

**Authors:** Alyssa Mooney, Aklilu Kidanu, Heather M Bradley, Evelyn Kuor Kumoji, Caitlin E Kennedy, Deanna Kerrigan

**Affiliations:** 1 Department of International Health, Johns Hopkins Bloomberg School of Public Health, 615 North Wolfe Street, Baltimore, MD, USA; 2 Miz-Hasab Research Center, Haile Gebre Selassie Road, Addis Ababa, Ethiopia; 3 3Centers for Disease Control and Prevention, Division of STD Prevention, 1600 Clifton Rd, NE, MS E-02, Atlanta, Georgia, USA; 4 Center for Communication Programs, Johns Hopkins University Bloomberg School of Public Health, Baltimore, MD, USA

**Keywords:** Sex workers, HIV/AIDS, Ethiopia, Condom use, Violence, Alcohol abuse

## Abstract

**Background:**

Although reported condom use between female sex workers and their clients is high in Ethiopia, condom use with regular, non-paying partners remains low, posing a substantial risk of HIV infection to sex workers, their partners and the general population. Previous studies have identified the synergistic effects of substance abuse, violence and HIV risk, but few have examined these inter-relationships among female sex workers and their regular, non-paying partners. This study explored the associations between work-related violence, alcohol abuse and inconsistent condom use among establishment-based female sex workers and their regular, non-paying partners in Adama City, Ethiopia.

**Methods:**

A cross-sectional survey was conducted with 350 establishment-based female sex workers, aged 15–35, at 63 bars, hotels and nightclubs. Multivariate logistic regression analysis was conducted to test the association between work-related violence and condom use with regular, non-paying partners, controlling for age, overall income, education and sex workers’ total number of sexual partners in the past week. Alcohol abuse was explored as an effect modifier.

**Results:**

Respondents reported a high prevalence of work-related violence (59%) and alcohol abuse (51%). Work-related violence was statistically significantly associated with unprotected sex with regular, non-paying partners among those who abused alcohol (OR: 6.34, 95% CI: 2.43-16.56) and among those who did not (OR: 2.98, 95% CI: 1.36-6.54). Alcohol abuse was not associated with inconsistent condom use within these partnerships, though it may strengthen the effect of work-related violence on unprotected sex.

**Conclusions:**

Findings suggest violence against establishment-based female sex workers is associated with HIV risk within regular, non-paying partnerships. Qualitative work is needed to better understand the links between a violent work environment and condom use with regular, non-paying partners and how interventions can be implemented in this context to prevent violence against sex workers and reduce HIV transmission.

## Background

The syndemic of substance abuse, violence and AIDS, initially conceptualized by Singer [1], has been studied extensively among impoverished, urban women in the United States [2]. In their review of the literature, Meyer et al. [2] found that women who experienced intimate partner violence faced barriers to negotiating condom use, that substance abuse was associated with increased violence and sexual risk-taking and that women who experienced violence were more likely to abuse substances. Despite the significance of substance abuse and violence in contributing to the burden of HIV, little research has examined its effects among female sex workers (FSWs) in countries with a high prevalence of HIV, particularly within their regular, non-paying partnerships.

Previous studies in the U.S. and China have identified associations between FSWs’ drug and alcohol abuse and experiences of physical and sexual violence perpetrated by both clients and regular, non-paying partners [3,4]. FSWs are subjected to high rates of physical and sexual abuse both prior to and during their engagement in sex work. Violence against FSWs may be perpetrated by regular, non-paying partners; clients; police; managers; and others [3-13]. Research has tended to look cross-sectionally at the coexistence of alcohol abuse and victimization, limiting the ability to determine causality. However, one longitudinal study in the U.S. [14] found that women’s alcohol abuse did not increase the odds of a new assault, but that experiencing a new assault significantly increased the odds of subsequent alcohol abuse by the victim.

The associations between FSWs’ experiences of violence and exposure to HIV risk within their client and regular, non-paying partnerships are well-documented. Studies have shown that FSWs who have ever been sexually or physically abused are more likely to report sexually transmitted infections (STIs) and inconsistent condom use with clients [5,15]. All types of violence (sexual, physical and emotional) perpetrated by clients or regular, non-paying partners have been associated with STIs [4]. In particular, the fear of victimization constituted a significant barrier to condom use negotiation [10,16-19].

There is a paucity of data on the links between violence and HIV risk within regular, non-paying partnerships in the developing world. One recent study in southern India examining the link between physical and/or sexual violence and inconsistent condom use found no association in non-paying partnerships, while observing a positive relationship in regular and occasional client partnerships [20]. A study in China examining physical, sexual and emotional violence found the reverse: all types of violence were associated with inconsistent condom use with regular, non-paying partners, but not with clients [4].

Other literature suggests the importance of lifetime gender-based violence (verbal, physical and/or sexual) among FSWs in relation to STIs and inconsistent condom use with clients and other partners [21], suggesting that experiences of violence impact women’s HIV risk beyond immediate partnerships. However, the link between FSWs’ violent work environments and HIV risk with regular, non-paying partners has not been investigated. The current study examined whether experiencing any physical, sexual or emotional violence in relation to one’s work was associated with inconsistent condom use in regular, non-paying partnerships.

Heavy alcohol consumption among FSWs has been documented globally [22]. Alcohol use by FSWs can be partially explained by its wide availability at venues where FSWs operate [23-26] and the fact that FSWs are often obliged to drink with clients [7,27-29]. Alcohol use by FSWs may also have a psychological component, and has been reported as a possible coping mechanism in several settings [5,8,30].

In addition to the effects on FSWs’ general mental and physical health, alcohol abuse has the potential to increase HIV risk in an already vulnerable population. Though FSWs operate in highly diverse environments, the association between alcohol use and increased odds of inconsistent condom use and STIs has been found in studies among FSWs in many developing countries [24,26,27,31-33]. In qualitative work conducted in Ethiopia and Cambodia, FSWs explained how alcohol use created a barrier to ensuring condom use with clients; it reduced their level of control [29] and intoxicated clients tended to refuse to use condoms [9,34].

Along the transport corridors of Ethiopia, the prevalence of HIV among women in the general population is 8.6%; among FSWs, it is 25.3% [35]. FSWs’ reported condom use with clients has increased dramatically over the past two decades, from 5.3% in 1989 to 99.4% in 2009, though condom use at last sex with regular, non-paying partners is significantly lower at 65.7% [36]. Although FSWs’ regular, non-paying partnerships sometimes begin as client relationships, a large discrepancy in condom use between the two partnership types has been identified in numerous studies worldwide [15,37-42]. In a qualitative study of FSWs in two Ethiopian towns, many participants reported that they did not deem it necessary to use condoms with sexual partners outside of their work [29].

Unprotected sex with regular, non-paying partners may put both FSWs and their partners at higher risk of HIV infection. For example, research in Benin found that regular, non-paying partners of FSWs had particularly large numbers of partners, concurrent partnerships with other FSWs, low rates of condom use, and higher HIV prevalence as compared to the new and regular clients of sex workers [43]. Studies on the risk of HIV within FSWs’ regular, non-paying partnerships in Ethiopia have not yet been conducted. However, research in Ethiopia has shown that these partnerships may arise from FSW-client relationships [29]. Demographic Health Survey data [44] indicate that HIV prevalence among clients of FSWs is substantially higher than in the general population (4.3% and 1.5%, respectively). Considering the high prevalence of HIV among Ethiopian FSWs [35] and their clients, the evolution of clients to regular, non-paying partners, and the low rate of condom use with the latter, it is likely that these partnerships present considerable risk.

While there is an increasing understanding of the synergistic effects of substance abuse, violence and HIV, little research has examined these associations as they relate to FSWs’ condom use with regular, non-paying partners, and to date, no such studies have been conducted in Ethiopia. Intimate partner violence is common in Ethiopia, with women’s reported lifetime prevalence ranging from 51-78% [45]. Qualitative work has indicated that in communities studied, intimate partner violence is considered acceptable under certain circumstances and social disincentives prevent reporting [46]. While research has suggested that FSWs in Ethiopia experience violence perpetrated by clients, establishment owners and other women [29], such work-related violence has not yet been examined as a contributor to HIV risk within regular, non-paying partnerships. This study aimed to investigate the influences of establishment-based FSWs’ alcohol abuse and experiences of violence on inconsistent condom use with regular, non-paying partners in Adama City, Ethiopia. Regular, non-paying partners are defined as boyfriends, husbands, or any other regular partners who do not pay directly for sex. Due to the high rate of consistent condom use with new and regular clients reported by respondents in this study (99.4%), we do not examine correlates of condom use within these partnerships.

## Methods

### Study design and data collection

Data collection was conducted by Miz-Hasab Research Center (MHRC), a licensed private research center based in Addis Ababa, Ethiopia’s capital. Data were collected between December 2009 and February 2010 using a structured survey questionnaire. FSWs were surveyed in Adama City, selected for its high prevalence of sex work, due to the city’s placement as a stop-over for truckers and businessmen traveling between the Port of Djibouti and Addis Ababa.

The survey was conducted with 350 FSWs in 63 establishments, including nightclubs, bars and hotels. Though sex work is illegal in Ethiopia, FSWs were typically employed by owners of licensed establishments as waitresses to increase drink sales and room rentals. Establishment owners charged clients for rooms, and FSWs generally paid money to establishment owners for time spent away from the venue with a client, or for breaking establishment rules.

A list of the total number of each establishment type was developed by the MHRC from a list of licensed establishments obtained from Health Communication Partnership and Elilta, two organizations that implement HIV prevention interventions with FSWs in Ethiopia. The list included establishments where at least five FSWs worked. Proportions of each establishment type were calculated from the total list of establishments, which were used to determine the proportion of the total sample of FSWs to select from each establishment type. The sample of establishments was randomly selected, and included approximately half of the establishments of each type. FSWs were selected from establishments using a convenience sample. Unlicensed venues were not included because FSWs generally did not drink at these establishments, where the ability to serve alcohol was limited. Often, only one to two FSWs were employed at these venues, and they were typically new to sex work and reluctant to participate in the study. Additionally, licensed venues where fewer than five FSWs operated were excluded.

Surveys took an average of 70 minutes, and were generally conducted in bedrooms or other private areas at participants’ work places after obtaining oral consent. Survey questions assessed FSWs’ demographic and socioeconomic characteristics, experience as a sex worker, relationships with establishment owners, patterns of alcohol use, sexual behavior, and condom use with various partner types. Six data collectors and two supervisors were selected from the MHRC staff, all of whom were trained for the study and had substantial previous experience in survey research.

### Ethical considerations

Ethical approval for the study was obtained from the Oromiya Health Bureau in Adama City. All participants provided informed consent.

### Outcome variables

Respondents were asked, “How often do you use condoms with regular, non-paying sex partners?” This was measured using a four-point Likert scale (always = 1, never = 4). A binary variable was created whereby sometimes, often or never were considered inconsistent condom use. No time period was specified.

### Independent variables

Respondents were asked, “In relation to your work, have you ever experience physical violence?” (yes/no). The same question was asked regarding physical danger, emotional abuse, threats and forced sex. Respondents were not asked to identify perpetrator types. A binary variable for work-related violence was developed and defined as a positive response to at least one of these five questions.

Alcohol abuse was measured using the four-item CAGE assessment (need to *cut* down on drinking, *annoyance* by critics, *guilt* about drinking, and *eye*-opening morning drinking). Two or more yes responses indicated alcohol abuse, as this cut-off has been shown to provide the best combination of specificity and sensitivity [47]. CAGE is a widely-used alcohol abuse screening tool, shown to have high test-retest reliability, adequate correlations with other instruments, and high validity in medical and surgical inpatients, psychiatric inpatients, and ambulatory medical patients [47]. While created for the U.S. population, the CAGE screening tool has been used successfully in studies of alcohol abuse in both urban and rural regions of Ethiopia, including FSW populations [31,48,49].

Additional socioeconomic and interpersonal factors which could potentially affect the outcome under study were identified from the literature and included in the analysis. These included age (15–19, 20–24, 25+), overall monthly income in Ethiopian Birr (0–999 ETB/0-57 USD, 1,000-2,999 ETB/58-171 USD, 3,000+ ETB/172+ USD), educational attainment (none, primary, secondary or higher), total number of sexual partners of any type in the past week (dichotomized at the median into 0–2 and 3+), sex work establishment type (bar, nightclub, hotel) and sex work duration (0–6 months, 7–36 months, 37+ months). Categories for age, income and sex work duration were created based on their distributions, using approximately the 25th and 75th percentiles as cut-offs to create three groups for each variable. Although previous studies have controlled for marital status and sex work as participants’ main occupation [3,50], we did not include these variables because none of the participants were currently married or co-habiting, and virtually all (98%) reported sex work as their main occupation.

### Data analysis

Statistical analysis was conducted using STATA version 12 software. Of the 350 respondents, 39 were excluded from the analysis because of missing data on the following variables: CAGE (32 missing observations), number of partners (4 missing observations), and the outcome of inconsistent condom use (3 missing observations). Bivariate analysis was used to assess the prevalence of alcohol abuse and work-related violence by demographic, socioeconomic and interpersonal characteristics. To determine if work-related violence mediated the effect of alcohol abuse on inconsistent condom use with regular, non-paying partners, simple logistic regressions were used first to test associations between alcohol abuse and work-related violence, alcohol abuse and inconsistent condom use, and work-related violence and inconsistent condom use. The relationship between alcohol abuse and inconsistent condom use with regular non-paying partners was not significant, so mediation analysis was not continued.

Multivariate logistic regression models were developed to test the association between work-related violence and inconsistent condom use with regular, non-paying partners among FSWs who did and did not abuse alcohol. Stratification was used to examine alcohol abuse as a moderator of the effect of work-related violence on inconsistent condom use. In addition, an interaction term was added to the unstratified model to test the interaction between alcohol abuse and violence, but did not produce a significant regression coefficient. However, the study may not have been adequately powered to detect an interaction, as this was done as an adhoc analysis.

Age, income, education and total number of partners of any type in the past week were selected a priori as important confounders based on previous studies of condom use among FSWs. Multi-collinearity was assessed using odds ratios of 2.5 as a cut-off, which indicated no collinearity among the variables of interest. The Akaike information criterion (AIC) was used to compare nested models. The first included work-related violence as the only independent variable; demographic and socio-economic variables (age, income and education) were added to the second; number of partners to the third; sex work duration to the fourth; and establishment type to the fifth. The Hosmer Lemeshow test was used to measure model fit (the finals model indicated adequate fit with p = 0.751 and p = 0.573 for alcohol abuse and no alcohol abuse models, respectively). Duration of sex work and establishment type were excluded from the final models because the variables were insignificant in bivariate and multivariate regressions and decreased model fit based on the AIC and Hosmer Lemeshow tests. Odds ratios and 95% confidence intervals were obtained for each variable.

## Results

### Sample characteristics

Demographic, socioeconomic and interpersonal characteristics of the sample are displayed in Table 1. Respondents had been involved in sex work for an average of 22.4 months (SD = 20.7 months), and nearly all (98%) reported that it was their main occupation. Twenty-five percent worked at bars, 26% at nightclubs and 49% at hotels. Mean number of total partners in the past week was three (SD = 1.8). The majority of respondents (70%) had never been married, and the remainder (30%) were divorced, widowed, or separated. Most (67%) did not have children. Sixty-five percent were primary school-educated, while 15% had no education and 20% had attained secondary education or higher. Mean monthly income was 1840 ETB/$105 (SD = 1355 ETB/$77) and mean age was 21.5 years (SD = 3.6 years).

**Table 1:** Demographic, socioeconomic and interpersonal characteristics (n = 311)

	**N**	**%**
Age		
15-19	101	32.5
20-24	144	46.3
25+	66	21.2
Monthly income in ETB 1 USD = 17.5 ETB		
<1000	87	28.0
1000-2999	168	54.0
3000+	56	18.0
Education		
None	47	15.1
Primary	203	65.3
Secondary or higher	61	19.6
Religion		
Orthodox	282	90.7
Muslim	22	7.1
Catholic, Protestant and other	7	2.2
Ethnic group		
Oromo	131	42.1
Amhara	108	34.7
Tigraway	24	7.7
Guragie	22	7.1
Welaita	12	3.9
Other	14	4.5
Marital status		
Never married	216	69.5
Divorced/separated/widowed	95	30.6
Children		
Yes	103	33.1
No	208	66.7
Number of children (among those with children)		
1	89	86.4
2+	14	13.6
Current main occupation		
Sex worker	304	97.8
Waitress	7	2.2
Sex work establishment type		
Bar	77	24.8
Nightclub	82	26.4
Hotel	152	48.9
	Mean	SD
Total partners in past week (all types)	3.0	1.8
Number of regular clients in past week	1.3	5.0
Number of new clients in past week	2.3	5.7
Months working in sex work	22.4	20.7
Months living in Adama	33.5	47.8

### Alcohol abuse, work-related violence and inconsistent condom use

Fifty-one percent reported alcohol abuse based on the CAGE assessment. When asked where they usually drank alcohol, nearly all (96%) reported primarily drinking in the workplace. Respondents primarily drank with new clients (49%) and regular clients (45%); just 2% reported primarily drinking with a boyfriend.

Any experience of violence in relation to one’s work was reported by 59% of respondents (threats were reported by 39%, physical violence by 33%, emotional abuse by 26%, physical danger by 17% and forced sex by 8%).

Thirty-eight percent of respondents reported inconsistent condom use with regular, non-paying partners, while only 0.3% reported inconsistent condom use with regular clients and 1% with non-regular clients. Table 2 displays the relationships between work-related violence, alcohol abuse and inconsistent condom use with regular, non-paying partners. Of those who reported violent experiences, 60% abused alcohol while 40% did not (p < .0001). Overall, inconsistent condom use was far more prevalent among respondents who experienced work-related violence (49.7%, as compared to 20.3%, p < .0001). Though inconsistent condom use was more prevalent in the alcohol abuse group than the reference group (42%, compared to 33%), the difference was not statistically significant, and therefore mediation analyses were not pursued. In addition, inconsistent condom use among those who experienced violence was not substantially higher among those who abused alcohol (52%, as compared to 47%).

**Table 2:** Relationships between work-related violence, alcohol abuse, and inconsistent condom use with regular, non-paying partners (n = 311)

	**Alcohol abuse**	**No alcohol abuse**	**Total**
**Work-related violence**	110	73	183
	*ICU: 51.8% (57)*	*ICU: 46.6% (34)*	*ICU: 49.7% (91)*
**No work-related violence**	48	80	128
	*ICU: 18.8% (9)*	*ICU: 21.3% (17)*	*ICU: 20.3% (26)*
**Total**	158	153	311
	*ICU: 41.8% (66)*	*ICU: 33.3% (51)*	*ICU: 37.6% (117)*

Note: *ICU* Inconsistent condom use with regular, non-paying partners.

Results from multivariate logistic regression analyses, stratified by alcohol abuse, are displayed in Table 3. Findings indicated inconsistent condom use with regular, non-paying partners was strongly associated with experiencing work-related violence in both the alcohol abuse group (OR: 6.34, 95% CI: 2.43-16.56) and reference group (OR: 2.98, 95% CI: 1.36-6.54) when controlling for age, education, income and number of total partners in the past week. Earning a middle or high income was also associated with the outcome in both groups (alcohol abuse group: middle income OR 8.33, 95% CI: 2.96-23.45, high income OR 8.51, 95% CI: 2.58-28.10; reference group: middle income OR 5.51, 95% CI: 1.62-15.76, high income OR 7.27, 95% CI: 1.63-32.52). No education was associated with the outcome in the alcohol abuse group (OR 6.94, 95% CI: 1.74-27.73). Though the effect of work-related violence on inconsistent condom use was stronger in the alcohol abuse group, the interaction between violence and alcohol was not statistically significant when an interaction term was added to the model without stratification.

**Table 3:** Multivariable logistic regression analysis of associations between violence and inconsistent condom use with regular, non-paying partners, stratified by alcohol abuse (n = 311)

	**Inconsistent condom use with regular, non-paying partners**	
	**Alcohol abuse** **OR (95% CI)**	**No alcohol abuse** **OR (95% CI)**
Work-related violence	6.34 (2.43-16.56)	2.98 (1.36-6.54)
Total partners (all types) in past week (≥3)	0.48 (0.22-1.06)	0.75 (0.35-1.63)
Age	--	--
15-19	1.00	1.00
20-24	1.17 (0.52-2.63)	1.11 (0.43-2.86)
25+	0.80 (0.23-2.81)	2.73 (0.99-7.52)
Monthly income (ETB) 1 USD = 17.5 ETB	--	--
<1000	1.00	1.00
1000-2999	8.33 (2.96-23.45)	5.51 (1.62-15.76)
3000+	8.51 (2.58-28.10)	7.27 (1.63-32.52)
Education	--	--
Secondary school or higher	1.00	1.00
Primary school	1.96 (0.81-4.73)	1.11 (0.37-3.35)
None	6.94 (1.74-27.73)	2.03 (0.53-7.79)

## Discussion

We examined the effects of alcohol abuse and violence on FSWs’ unprotected sex with regular, non-paying partners, relationships which may present a high risk of HIV transmission yet which have not yet been examined in the Ethiopian context. In our survey, 38% of FSWs reported inconsistent condom use with regular, non-paying partners. The majority (59%) reported experiencing work-related violence, and 51% reported alcohol abuse. Findings indicated that inconsistent condom use with regular, non-paying partners is associated with experiences of work-related violence and higher income. No significant relationship was found between alcohol abuse and inconsistent condom use within these partnerships. Data suggested that alcohol abuse among FSWs in Adama City is highly contextualized, and drinking occurs almost exclusively with clients and in work settings, so may not have a substantial effect on sexual behavior within regular, non-paying partnerships.

### Work-related violence and inconsistent condom use with regular, non-paying partners

Our findings suggest there may be a relationship between experiencing violence at work and being victimized by regular, non-paying partners. In studies of FSWs in New York, South Africa, and China, participants reported high rates of violence perpetrated by both clients and regular, non-paying partners [3-5], and in China there was a correlation between the two [4]. FSWs reporting work-related violence may have been more likely to engage in violent non-paying partnerships, which then contributed to inconsistent condom use due to fear of victimization.

However, it is unclear why work-related violence did not interfere with condom use in new or regular client partnerships, which was reported to be consistent by over 99% of respondents. This could indicate that condom use with new and regular clients was over-reported, or that client violence shaped behavior less than violence by regular, non-paying partners. This result is consistent with previous research in China which found that violence perpetrated by regular, non-paying partners was associated with inconsistent condom use with partners of that type, but abuse by clients was not related to condom use with clients [4]. STI history among FSWs was, however, associated with violence perpetrated by both partner types [4].

Although this study distinguished between clients and regular, non-paying partners, FSWs may not perceive a clear distinction. Previous research in Madagascar found that some FSWs described sexual relationships that transitioned more fluidly across categories, with client relationships evolving into regular, non-paying partnerships and back [51]. In Kenya, FSWs were unable to clearly distinguish between clients and regular, non-paying partners; after having sex several times, FSWs identified clients as boyfriends, yet they still paid for sex a significant proportion of the time [41]. These fluid categories may offer one possible explanation for our findings: if there is overlap between clients and regular, non-paying partners, work-related violence reported in our study could be committed by the latter. Perhaps as clients become regular, non-paying partners and the relationships take on higher levels of intimacy, participants’ continued sexual interactions with clients result in jealousy, threats and acts of violence committed by these regular, non-paying partners; these have been identified as motivations for violence among FSWs in India and Madagascar [7,51]. A combination of fear of victimization and increasing levels of relationship intimacy may lead to inconsistent condom use with regular, non-paying partners as a strategy to appease partners and de-escalate violent episodes.

### Higher income and inconsistent condom use with regular, non-paying partners

We identified a significant relationship between inconsistent condom use with regular, non-paying partners and higher income. Evidence suggests FSWs may receive more money from regular, “non-paying” partners than clients, and that they may be willing to sacrifice condom use to ensure the stability of this income [34,41]. In a study of FSWs in Kenya, men termed “regular partners” in fact paid one-half to three-quarters of the times they had sex, paid significantly more than clients, and provided indirect financial and material support [41]. Similarly, a study in India found that most FSWs received economic support from regular, non-paying partners [50]. A study of FSWs in Indonesia found that regular clients frequently took FSWs as mistresses and provided them with steady economic support [52]. FSWs in these relationships tended to reduce their numbers of partners, which resulted in them feeling protected against HIV and thereby influencing a decrease in their use of condoms as the partnerships became more familiar and intimate.

It is quite possible that FSWs who participated in our study were receiving significant monetary support from their regular, non-paying partners, which may explain the association between higher income and inconsistent condom use with these partners. It should also be noted that types of income were not distinguished in the analysis, and some income may have been generated from jobs outside of sex work. We do not expect that other types of income would have confounded results, as only 7% reported working in any job outside of sex work, and 98% reported that sex work was their main occupation.

### Alcohol abuse and inconsistent condom use with regular, non-paying partners

Fifty-one percent of respondents reported alcohol abuse, measured by two or more positive responses to the CAGE assessment. Alem et al. [31] used the same measure and found a lower rate of alcohol abuse in their study of FSWs in urban Ethiopia (31%), though this could be a result of their inclusion of street-based FSWs who may drink less frequently than FSWs working at establishments where alcohol is served.

Findings from the current study did not indicate a significant association between alcohol abuse and inconsistent condom use with regular, non-paying partners, and suggested alcohol abuse occurs primarily with clients in a work setting. Though alcohol abuse did appear to strengthen the effect of work-related violence on inconsistent condom use, interaction was not significant. Based on our findings, it appears that violence has a greater influence than alcohol abuse on unprotected sex with regular, non-paying partners among establishment-based FSWs in Adama City. However, the high rate of reported alcohol abuse in our sample has important health implications which should be addressed.

Though not investigated in our analysis, other research has found that feelings of trust and intimacy, the desire to separate one’s work from private relationships, and the perception that familiar partners are lower risk, also play a large role in FSWs’ inconsistent condom use with regular, non-paying partners [37,39,52,53]. Kerrigan et al. [54] and Murray et al. [55] found that this relationship extends to clients, as perceived intimacy with regular clients is strongly associated with lower condom use. We did not have sufficient power to examine correlates of condom use with regular clients, which was reported to be consistent by 99.7% of respondents.

The major limitation to this study was its cross-sectional design, which did not allow causal inferences to be made regarding experiences of violence and sexual risk behavior. Additionally, the measures used were limited in several ways. Measures of work-related violence did not specify who perpetrators were and whether they included regular, non-paying partners, which would clarify the relationship between work-related violence and inconsistent condom use with these partners. Violence, condom use and alcohol abuse variables lacked time frames.

Additionally, the CAGE assessment for alcohol abuse was developed for use in the U.S. and may not necessarily be a valid measure in this geographic region or population. Even within the U.S., limitations to its validity have been identified in specific populations [47]. Though greater use of standardized measures in studies of alcohol use among FSWs is needed, an assessment of alcohol abuse in general may not be the best measure to examine the association between alcohol use and HIV risk, as it does not take into account whether alcohol consumption is occurring at the time of sexual encounters. Drinking in the context of such encounters may have a stronger relationship with sexual risk behavior than does the frequency or quantity of alcohol use [56].

### Implications of research

Experiences of violence are common among establishment-based FSWs in Adama City. While the impact violence has on women’s immediate physical and emotional health is clear, this study suggests these experiences are associated with increased HIV risk within FSWs’ regular, non-paying partnerships as well, and structural interventions to prevent violence are needed. Recommendations for HIV prevention interventions for sex workers in low- and middle-income countries released by the World Health Organization (WHO) in 2012 [57] suggest collaboration between policy-makers, religious and public leaders, civil society and FSWs’ organizations to develop policies and services that protect FSWs from violence and facilitate reporting and redressal. These may include measures such as establishing antidiscrimination laws, legal literacy and legal and support services for FSWs who experience violence, and training law enforcement, health and social service providers to uphold and ascribe to FSWs’ human rights [57].

The nature of FSWs’ regular, non-paying partnerships in this context, and how such partnerships intersect with FSWs’ experiences of violence and associated HIV risk should be further explored. Research may seek to further characterize regular, non-paying partnerships in this setting, such as typical numbers of regular, non-paying partners; varying partner types within this category; relationship durations; whether partnerships arose from client or supervisor relationships; and patterns of violence and socio-economic exchange within these partnerships. Additional information about these characteristics of relationships could improve understanding of associations with inconsistent condom use.

Developing standardized measures for violence in various FSW populations, which would account for diverse types of violence and perpetrators, would be useful to ensure consistent interpretation by respondents, allow for comparison across studies and support a deeper understanding of the relationship between violence and inconsistent condom use among FSWs across partner types.

## Conclusions

Establishment-based FSWs in Adama City, Ethiopia experience high rates of violence and alcohol abuse. Cross-sectional analysis showed that work-related violence and higher income were associated with inconsistent condom use with regular, non-paying partners. Alcohol abuse was not associated with condom use within these partnerships, though it may strengthen the effect of violence on unprotected sex. Sex establishment working environments have been shown to affect the risk of HIV and violence experienced by FSWs in many settings. However, structural interventions to reduce violence against FSWs for HIV prevention have been rare, despite the high prevalence of violence in this population [15]. Findings from this study suggest a need for integrated HIV and violence prevention programming among FSWs to reduce HIV-related risk in this population.

## Competing interests

The authors declare that they have no competing interests.

## Authors’ contributions

AK, HMB, and EKK, designed the study. AK and HMB trained data collectors and AK managed data collection. AM conducted the statistical analysis and drafted the manuscript. CEK and DK provided input into data analysis and overall project management. All authors reviewed, edited and approved the final manuscript.

## Pre-publication history

The pre-publication history for this paper can be accessed here:

http://www.biomedcentral.com/1471-2458/13/771/prepub
